# Sulfur dioxide resistance in *Saccharomyces cerevisiae*: beyond *SSU1*

**DOI:** 10.15698/mic2019.12.699

**Published:** 2019-11-21

**Authors:** Estéfani García-Ríos, José Manuel Guillamón

**Affiliations:** 1 Food Biotechnology Department, Instituto de Agroquímica y Tecnología de Alimentos (IATA), Consejo Superior de Investigaciones Científicas (CSIC), Valencia, Spain.

**Keywords:** Saccharomyces cerevisiae, wine, sulfur dioxide, Com2

## Abstract

Sulfite resistance is an important oenological trait for wine yeasts because this compound is used during winemaking as a microbial inhibitor and antioxidant. The molecular mechanisms by which *Saccharomyces cerevisiae* responds and tolerates SO_2_ have been mainly focused on the sulfite efflux pump encoded by *SSU1*. Different chromosomal rearrangements in the regulatory region of this gene have been correlated with improved sulfite tolerance. However, other molecular factors must contribute to this trait because the *SSU1* gene activity does not always fit with sulfite tolerance. An interesting approach to shed light onto this issue could be found by Lage *et al.* (2019). These authors have combined transcriptomic and genome-wide analysis to describe how the poorly characterized transcription factor Com2 controls, directly or indirectly, the expression of more than 80% of the genes activated by SO_2_. Additionally, large-scale phenotyping revealed the identification of 50 Com2-targets contributing to the protection against SO_2_. This information is very interesting for gaining knowledge regarding this important industrial trait.

Microorganisms utilize a great variety of genetic strategies to adapt to natural and human-made environments. The wine strains of *S. cerevisiae* are a clear example of highly specialized microorganisms that have evolved to use the different ecological niches provided by human activity. The specific genetic characteristics of the wine yeast strains are a consequence of the process of domestication [1–6]. Thus, in the age of high throughput sequencing technologies and omics data, a current challenge is to unveil the molecular determinants underlying a specific trait of industrial interest. This knowledge will be of paramount importance for selection and genetic improvement of the industrial strains.

Sulfite (SO_3_^2−^), which is produced by dissolution of sulfur dioxide (SO_2_) in water, is used during winemaking as a microbial inhibitor and antioxidant. Therefore, sulfite resistance is a desired trait for wine yeast strains [7]. *S. cerevisiae* cells have different mechanisms to deal with the stress produced by sulfites including the increase in the production of acetaldehyde, which binds to SO_3_^2−^, the regulation of the sulfite uptake pathway, and sulfite efflux through a plasma membrane pump encoded by the *SSU1* gene [8]. Indeed, wine *S. cerevisiae* strains are considerably more tolerant to SO_2_ than laboratory strains and the main molecular mechanism connected with this higher resistant phenotype results from a higher transcription of the *SSU1* gene. Among this group of strains, three chromosomal rearrangements (VIIItXVI, XVtXVI and Inv-XVI) have been described to up-regulate *SSU1* expression and thereby increasing sulfite tolerance [9–12]. In all cases, the chromosomal rearrangement involves the *SSU1* promoter and leads to its transcriptional up-regulation. Surprisingly, *SSU1* transcript levels are not responsive to SO_2_ [9, 10, 12], indicating that the different levels of resistance in *S. cerevisiae* wine strains are, in general, explained by the basal transcript levels of *SSU1*, except for the 71B strain that harbors a sulfur-inducible *SSU1* gene that may have gained a new regulatory system [13]. However, in some cases *SSU1* mRNA levels did not correlate with sulfite tolerance probably due to the contribution of other factors to yeast sulfite resistance. Thus, a key question remains to be addressed in the field: Which are the molecular mechanisms that explain yeast sulfite tolerance? This question is of paramount importance for understanding much better the regulation network between gene activity and metabolic response. However, this information is also crucial for the wine industry, which is leading a process of sulfite reduction in their wines

In this scenario, Lage *et al.*, (2019) [14] have recently demonstrated the involvement of Com2 as the main transcriptional factor regulating genes that impart SO_2_ resistance. The results clearly highlight Com2 as the main player in the reprogramming of yeast genomic expression under SO_2_ stress, regulating approximately 80% of the SO_2_-activated genes. Moreover, the involvement in SO_2_ tolerance of genes regulated by Com2 was validated by a large-scale phenotyping of the corresponding mutants, proving the susceptibility of these mutants to SO_2_. **Figure 1** shows a summary of the main mechanisms involved in SO_2_ resistance in *S. cerevisiae*.

**Figure 1:**
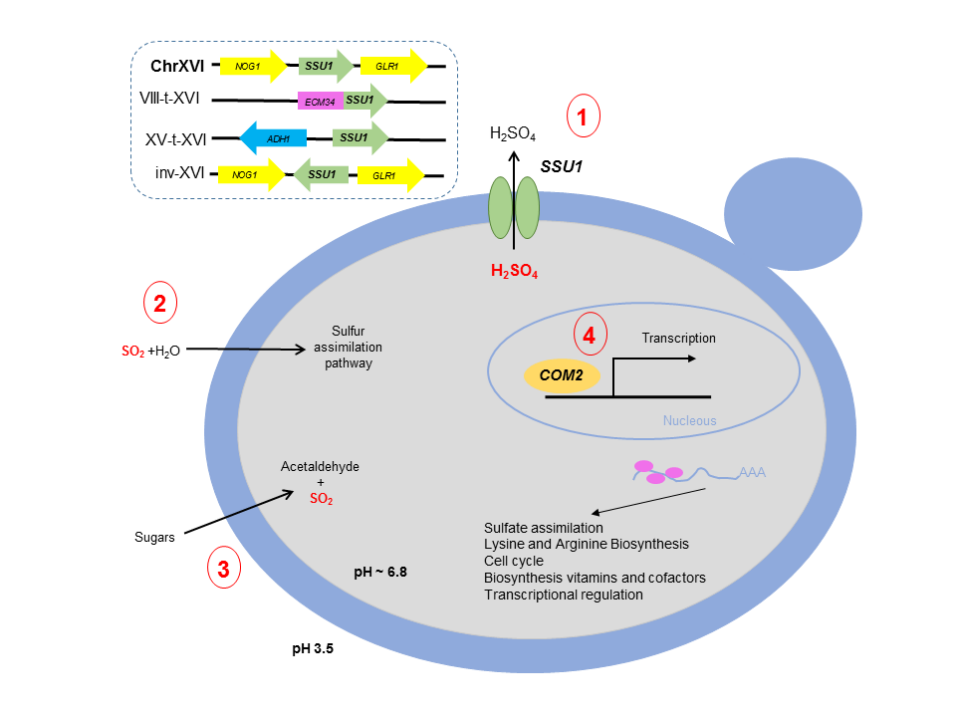
FIGURE 1: Schematic representation of the main mechanisms involved in sulfites resistance in the yeast *Saccharomyces cerevisiae*. (1) Efflux of SO_2_ mediated by *SSU1* including the different chromosomal rearrangements described so far; (2) Incorporation into the sulfur assimilation pathway; (3) Acetaldehyde production and (4) Com2 regulon.

*COM2* encodes an orphan homologue of the environmental stress-responsive transcription factors Msn2 and Msn4 [15] and, the genes regulated by Msn2 were also found to be regulated by Com2, suggesting some overlap between them. In a phenotypic assay with and without sulfite, the strain lacking the Com2 gene was more sensitive to sulfite than its wild-type counterpart (BY4741). The transcriptomic profiling of BY4741 and BY4741_*com2*? cells revealed dramatic changes in both strains especially after exposure to SO_2_ (0.5 mM), although this effect was markedly different in the two strains. A higher number of genes were differentially expressed in the wild type compared to the *com2*? strain, indicating that these SO_2_-responsive genes present in the wild type and without activation in the mutant strain could be considered as Com2 targets. No significant effect of Com2 was found in the absence of SO_2_. The functional categories obtained after analyzing these genes are old known from the transcriptomic analysis performed to date and are mainly related to nitrogen, sulfur and some aminoacids metabolism [16]. In this line, authors have shown that supplementation of the medium with arginine and lysine somehow alleviated SO_2_ toxicity. A plausible explanation could be that exposure to SO_2_ can lead to a depletion of intracellular lysine and arginine or, in the case of arginine, by contributing to the integrity of the cell wall and the plasma membrane [17].

However, as mentioned above, the exposition to SO_2_ during hundreds of years and thousands of generations have caused *S. cerevisiae* wine strains to be more tolerant to this compound than the laboratory strains. They have evolved to employ various anthropic niches or environments during the so-called “unaware domestication” process [1, 3, 18]. Their genomes present genetic polymorphisms with different evolutionary consequences all of which help wine yeast genomes to adapt [19, 20]. Thus, the main limitation of this study is the use of a single laboratory strain genetic background to investigate a phenotype of industrial relevance in the wine industry. Thus, the remaining question is: How much is the contribution of the Com2 in a wine yeast background adapted to sulfite stress? The key role of this gene in the laboratory strains is undeniable but we have to be careful when transferring this information from one background to another and, therefore, more studies are needed to determine its possible application in the industry.
